# Cognitive behavioural group therapy for male perpetrators of intimate partner violence: a systematic review

**DOI:** 10.1186/s12888-019-2010-1

**Published:** 2019-01-08

**Authors:** Merete Berg Nesset, Mariela Loreto Lara-Cabrera, Therese Kristine Dalsbø, Sindre Andre Pedersen, Johan Håkon Bjørngaard, Tom Palmstierna

**Affiliations:** 1 Forensic Department and Research Centre Brøset, St. Olav’s University Hospital, PO 1803 Lade, N-7440 Trondheim, Norway; 2 Faculty of medicine and health sciences, dept. of Mental Health, Norwegian University of Science and Technology (NTNU), Trondheim, Norway; 3 Tiller Community Mental Health Centre, Division of Psychiatry, St. Olav’s University Hospital, Trondheim, Norway; 4 Department of Research and Development, Division of Mental Health, St Olav’s University Hospital, Trondheim, Norway; 5 Norwegian Institute of Public Health, Oslo, Norway; 6 Library Section for Medicine and Health Sciences, NTNU University Library, NTNU – Norwegian University of Science and Technology, Trondheim, Norway; 7 Department of Social Science, Norwegian University of Science and Technology (NTNU), Trondheim, Norway; 8 Department of Clinical Neuroscience, Centre for Psychiatric Research, Karolinska Institutet, Stockholm, Sweden

**Keywords:** Batterer, CBT, Cognitive therapy, Group therapy, Intimate partner violence, Mental health, Perpetrator, Randomized controlled trials, Systematic review

## Abstract

**Background:**

Violence against intimate partners is a worldwide public health problem. Cognitive behavioural therapy delivered in a group format is widely used for the treatment of men’s violent behaviour towards their female partners. A Cochrane review about the effectiveness of this therapy from 2011 revealed a lack of controlled studies. Our aim is to update the current evidence on the effectiveness of cognitive behavioural group therapy on men’s violent behaviour towards their female partner.

**Methods:**

The Cochrane Library, the Campbell Collaboration Social, MEDLINE, PsychINFO, CINAHL, SCOPUS, Embase, Open Grey, Grey Literature Report, and Sociological Abstracts were searched for studies investigating the effectiveness of cognitive behavioural group therapy on intimate partner violence published in the period of January 1, 2010, to February 12, 2018. Manual searches were also performed to identify randomized and non-randomized controlled trials. Data extraction was done in duplicate. The primary outcome was the reduction in violent behaviour, and secondary outcomes were physical health, mental health, quality of life, emotion regulation, and substance use. Study quality was assessed with the Cochrane Collaboration’s risk of bias tool and the Risk of Bias In Non-Randomized Studies of Interventions tool. A narrative summary was used to describe the review findings.

**Results:**

We identified six new studies that met the inclusion criteria: four randomized controlled trials and two non-randomized trials. Three of the randomized controlled trials found a reduction in intimate partner violence after treatment. The fourth randomized trial found that a subsample of responding partners reported a reduction in violence but no changes in the men’s self-reported violence after treatment. No effect could be detected in the two non-randomized studies. Analysis of risk of bias revealed mixed results, indicating both strengths and weaknesses.

**Limitations:**

Only a limited amount of studies which scored as “low quality” were available.

**Conclusions:**

There is still insufficient evidence to confirm that cognitive behavioural group therapy for perpetrators of intimate partner violence has a positive effect. Future research should focus on randomized controlled studies distinguishing between convicted and non-convicted populations where violent behaviour is the primary outcome.

**Trial registration:**

CRD42016041493.

**Electronic supplementary material:**

The online version of this article (10.1186/s12888-019-2010-1) contains supplementary material, which is available to authorized users.

## Background

Intimate partner violence is a violation of human dignity and rights and includes various forms of physical, sexual, and psychological abuse [1–4]. In contrast to other types of violent acts, violence by an intimate partner often reoccur within the relationship and can go on for years [3, 5], and recidivism rates of 21% [6] to 42% [7] are reported. Violence against women is a global public health problem and studies on intimate partner violence suggest that nearly one third of women experience physical or sexual violence from an intimate partner during their lifetime [8]. Furthermore, WHO [9] and others [10] estimated that as many as 38% of female homicides globally were committed by male partners, and the global life-time prevalence of physical and/or sexual violence by an intimate partner was 30%. In addition, 20–75% of women have reported experiencing emotional violence [11].

Cognitive Behavioural Therapy (CBT) is one of the most actively researched psychotherapies and has received consistent empirical support for a host of mental health problems and conditions [12, 13]. In the treatment of aggressive behaviour, CBT interventions are now a commonly used approach to help different populations to regulate anger and aggressive behaviour [14]. The main techniques used in CBT focus on establishing a therapeutic relationship, behavioural change strategies, cognitive restructuring, modification of core beliefs and schemas, and the prevention of relapse and recurrence. Cognitive theory suggests that psychopathology is characterized by the activation of a conglomerate of related or contiguous dysfunctional beliefs, meanings, and memories that operate in coordination with affect, motivation, behaviour, and physiological responses [12]. Different psychopathological conditions are associated with specific biases that influence how an individual incorporates and responds to new information [12, 13].

CBT is commonly used to address dysfunctional anger and violent behaviour among intimate partners. Research on the effectiveness of such interventions has yielded mixed results [15, 16]. A systematic review in 2007 identified six studies (*N* = 2343) which consisted of a mix of convicted and non-convicted male participants [16]. One study (*N* = 218) compared feminist-cognitive-behavioural-group-therapy with process-psychodynamic group therapy [17]. The second study (*N* = 64) compared a 12-week CBT-based substance abuse and domestic violence group with a 12-week twelve-step facilitation group [18]. The results were inconclusive in each of the two studies. The other four studies compared CBT with no intervention (1771 participants in total) [19–22]. Only one of these showed a statistically significant effect in favour of CBT [22]. A meta-analysis showed that the relative risk for violence was 0.86 in favour of the intervention group with a confidence interval of 0.54–1.38. However, a combination of a low effect size and a wide confidence interval led to the conclusion that there was insufficient evidence concerning the effectiveness of CBT. A revision of this study in 2011 failed to identify new randomized controlled trials, precluding any new meta-analyses [23].

The primary aim of this systematic review is to examine new evidence for the effectiveness of group-based CBT on men’s violent behaviour towards their female partners. Secondly, we also review whether cognitive behavioural group therapy (CBGT) affects changes in self-reported physical health, mental health, quality of life, emotional regulation, substance use, and socioeconomic outcome among perpetrators.

## Methods

The systematic review was registered in the International Prospective Register of Systematic Reviews (PROSPERO), no: PROSPERO 2016:CRD42016041493, and conducted according to the Preferred Reporting Items for Systematic Reviews and Meta-Analyses (PRISMA) standards [24, 25].

### Eligibility criteria

1. Adult male participants aged 18 years or older who had a history of physical, psychological, or sexual violence towards their female intimate partners.
2. Participants voluntarily referred or convicted to treatment.
3. Studies examining the effect of cognitive behavioural group therapy.
4. The control group condition should be classified as applying no intervention, another intervention, or a waiting list.
5. The study should report on type, frequency and recurrence of physically, psychologically and/or sexually violent behaviour.
6. Eligible studies were required to be randomized or non-randomized controlled studies published in peer-reviewed journals during the publication period of January 1, 2010, to February 12, 2018.
7. The studies were written in English, Spanish, or Portuguese.
8. Studies examining perpetrators of human trafficking, child exposure to intimate partner violence, or dating violence among adolescents were excluded. Also, studies examining other forms of therapy than cognitive behavioural group therapy (i.e. couple’s therapy, individual therapy) were excluded.

### Search strategy

A systematic literature search was conducted with the assistance of a medical research librarian (S.A.P) on various databases: the Cochrane Library, the Campbell Collaboration Social, MEDLINE, PsychINFO, CINAHL, SCOPUS, Embase, Open Grey, Grey Literature Report, and Sociological Abstracts. The queries involved a combination of thesaurus and free-text terms that were optimised to identify studies on intimate partner violence and cognitive therapy in the respective databases (see additional file 1), building on a search strategy described by Smedslund et al. [16]. The search was limited to the period of January 1, 2010, to February 12, 2018, in order to find studies published since the review by Smedslund et al. [23]. In addition to examining the reference lists of included studies, the Journal of Interpersonal Violence and Journal of Family Violence were searched by hand for the relevant period.

### Data extraction

Two authors (M.B.N and M.L.L-C) independently screened the abstracts and titles of the retrieved references and assessed the full text of potentially eligible studies. Discrepancies were resolved by discussion with a third author (T.P). Two authors (M.B.N and M.L.L-C) extracted data from all included articles by following the Template for Intervention Description and Replication (TIDIeR) [26]. The items extracted and recorded were the study design, setting, sample characteristics like age, voluntarily or court-ordered to treatment, as well as outcomes, treatment fidelity and length of follow-up. Moreover, type of intervention, type of control condition, measurement tools, and timing of the outcome assessment. The predefined secondary outcomes were also recorded. We contacted authors for further information if needed. The final decisions on which studies that met the inclusion criteria were made after discussion among the review authors.

### Quality assessment

To determine the validity of randomized trials, three authors (M.B.N, M.L.L-C & T.D) worked independently using the Cochrane Collaboration’s risk of bias tool [27]. The same authors assessed the remaining studies using the Risk of Bias In Non-randomized Studies of Interventions (ROBINS-I) tool [28]. This process was followed by a discussion between all authors about the methodological quality of the included studies.

## Results

### Search results

**Fig. 1:**
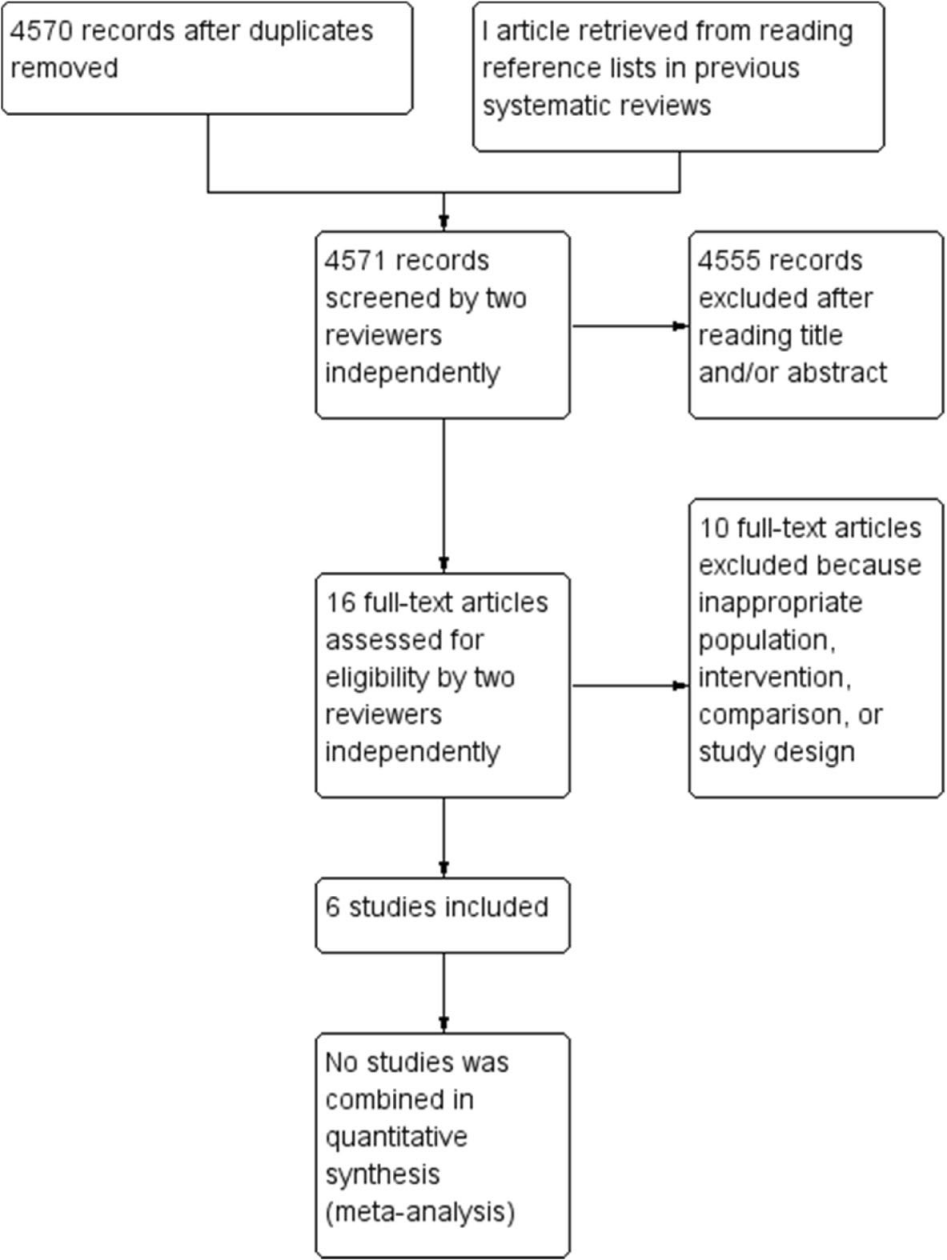
Flow diagram depicting the stages in the study selection process

The database searches yielded 4570 unique references (see Fig. 1, study flow diagram depicted from RevMan) [29]. Hand searching of the bibliographies of the systematic reviews and articles selected for the full text review revealed one additional study with potential relevance [30]. The full text of 16 articles was retrieved and reviewed in detail. One of these studies was excluded because it investigated the effect of individual therapy [31], while another was excluded because it investigated couples’ therapy [32]. One was excluded because it did not measure violent behaviour but rather thoughts and aggressive feelings [33], and four additional studies were excluded because the main intervention was not group CBT [30, 34–36].

### Characteristics of included studies

A total of six studies were finally included in the study following the screening process. Table 1 presents the characteristics of the four randomized controlled trials with 731 participants [37–40]. Table 2 presents the characteristics of the two non-randomized studies with 854 participants, one was a controlled retrospective cohort study [41], while the other was a quasi-experimental study [42]. The studies were conducted in Norway [39], United States [37, 38, 40], Sweden [41], and Spain [42] and published in English except for the study by Boira et al. [42], which was published in Spanish. The interventions described were carried out within special health services, a community setting serving victims and perpetrators of domestic violence, a prison or probation service setting, and a university setting.

**Table 1:** Characteristics of randomized controlled trials examining the effect of cognitive behaviour group therapy

Study, year, country	Setting	Population (N, mean age)	Intervention	Control condition	Outcome definition	Length of follow-up	Results: primary outcome
Alexander et al. [26], 2010, United States	Community setting serving victims and perpetrators of domestic violence	Male perpetrators (96.1% court-ordered) (*N* = 528, mean age 34.18 years)	Motivational Interviewing combined with Cognitive behavioural group therapy (SOCMI) 26 weeks	Cognitive behavioural group therapy (gender re-education), 26 weeks	CTS2, (psychological and physical aggression)	Perpetrator performed self-reports at 26 weeks’ post-treatment. Partner assessments were performed at 6 and 12 months	No changes in self-reported violence. Significant reductions in partner reports of physical violence at follow-up in the SOCMI group
Murphy et al. [28], 2017, United States	A community-based domestic violence agency	Male perpetrators (N = 42, mean age 34.38 years)	Cognitive behavioural therapy, 20 individual sessions (ICBT)	Cognitive behavioural group therapy (CBGT), 20 weekly 2-h sessions	CTS2 (physical, psychological aggression, emotional abuse, relationship adjustment)	Perpetrator and partner performed self-reports at baseline and 3, 6, 9 and 12 months after baseline	CBGT produced equivalent or greater benefits than ICBT. Significant reductions in self-reported violence across conditions, with no between condition differences. Partner reports revealed more favourable outcomes for group treatment on measures of physical and psychological violence
Palmstierna et al. [25], 2012, Norway	Specialised outpatient mental health service	Male perpetrators voluntarily seeking therapy (*N* = 26, mean age 35.00 years)	Cognitive behavioural group therapy. 15 weeks 2 h sessions	Waiting list	CTS extended version (physical, material, any violence, verbal aggression)	Assessment after 15 weeks of treatment and after 15 weeks on waiting list as compared to baseline assessment	Significant reductions in self-reported violence in treatment group as compared to the waiting list group
Taft et al. [29], 2016, United States	Veteran Affairs hospitals Clinician-referrals, self-referrals, court-referrals	Male perpetrators; military veterans or service members (*N* = 135, mean age 37.85 years)	Cognitive behavioural group therapy, 12 weekly 2 h sessions (trauma-informed group intervention)	Treatment as usual	CTS2 (physical assault, psychological aggression) MINI, CAPS, MMEA	Perpetrators performed self-reports at baseline and 3 and 6 months after baseline. Partner assessments were performed at baseline and after 3 and 6 months	Significantly greater reductions in reported physical and psychologically intimate partner violence in the intervention group, self- and partner reports combined

*CTS2* Conflict Tactics Scales–Revised, *CTS* Conflict Tactics Scales extended version, *MINI* The Mini-International Neuropsychiatric Interview, *CAPS* The Clinician-Administered PTSD Scale, *MMEA* Multidimensional Measure of Emotional Abuse. *CBGT* Cognitive Behaviour Group Therapy, *ICBT* Individual Cognitive Behaviour Therapy, *SOCMI* Stages-Of-Change Motivational Interviewing

**Table 2:** Characteristics of non-randomized studies examining the effect of cognitive behavior group therapy

Study, year, country	Setting	Population (N, mean age)	Intervention	Control condition	Outcome definition	Length of follow-up	Results: primary outcome
Haggård et al. [31], 2017, Sweden	Prison and probation offices	Consecutive sample of male IPV perpetrators: (*N* = 792, mean age 39.55 years)	Manual-based group program for male perpetrators (IDAP), including a pro-feminist psychoeducational approach	Concomitant IPV offender controls who did not enter IDAP	Any new convictions for any violent recidivism and IPV during the follow up time	From time of recruitment unto study (2004–2007) until March 2, 2011. Mean time at risk, 4.6 years	19% (*N* = 65) of IDAP participants and 19% (*N* = 84) controls recidivated in violence against a partner or former partner
Boira et a. [32], 2013, Spain	Setting unclear. Treatment delivered by psychologists specialized in intimate partner violence	Male perpetrators convicted for IPV and court ordered to treatment (*N* = 62, mean age 39.70 years)	Three treatment modalities: 1. Structured group 2. Unstructured group (open group format) 3. Individual therapy	Waiting list	Police reports on new intimate partner violence	18 months	6.4% of the participants across the interventions were reported to the police for new intimate partner violence

*CBT* Cognitive Behavioural Therapy*, IDAP* Integrated Domestic Abuse Program, *IPV* Intimate Partner Violence

The participants were recruited voluntarily or court-referred for treatment. Most of the participants in the studies were convicted of intimate partner violence. However, there were large notable differences concerning participant samples between the studies, ranging from 26 to 528 in the randomized controlled studies and between 62 and 792 in the non-randomized studies. The mean age of participants ranged from 34 to 40 years old.

The interventions used in the studies varied in content, length and how they were delivered. Palmstierna et al. [39] investigated the effect of cognitive behavioural group therapy (CBGT) delivered in a combination of three to four individual sessions followed by 15 two-hour group sessions. Alexander et al. [37] investigated the effect of 26 sessions of standard CBGT gender re-education. Murphy et al. [38] investigated the effect of 20 weekly 2-h sessions CBGT. Taft et al. [40] investigated the effect of 12 weekly 2-h sessions of trauma informed group intervention.

With regard to the non-randomised studies Haggård et al. [41] investigated the effect of an integrated domestic abuse program (IDAP) consisting of a minimum of 8 individual sessions and 27 two-hour group sessions, while Boira et al. [42] investigated the effect of a 20-session manualised CBGT-program.

In all studies, the group leaders were therapists trained on intervention with perpetrators of intimate partner violence (psychologists, doctoral students in clinical psychology, clinical psychology graduate student trainee, social workers, mental health nurses, or others with a university degree in behavioural science). The control groups were based on usual care [40, 41], an alternative intervention [37], a waiting list [39], or a comparison of the intervention with an open group format, individual therapy, or a waiting list [42], 20 sessions of standard individual cognitive therapy [38]. The intervention fidelity was measured in one study [37] by a blinded rater who listened to randomly selected audiotapes. Two studies reported treatment fidelity by recording group sessions followed by supervision to the instructors [38, 41].

### Quality assessment

**Fig. 2:**
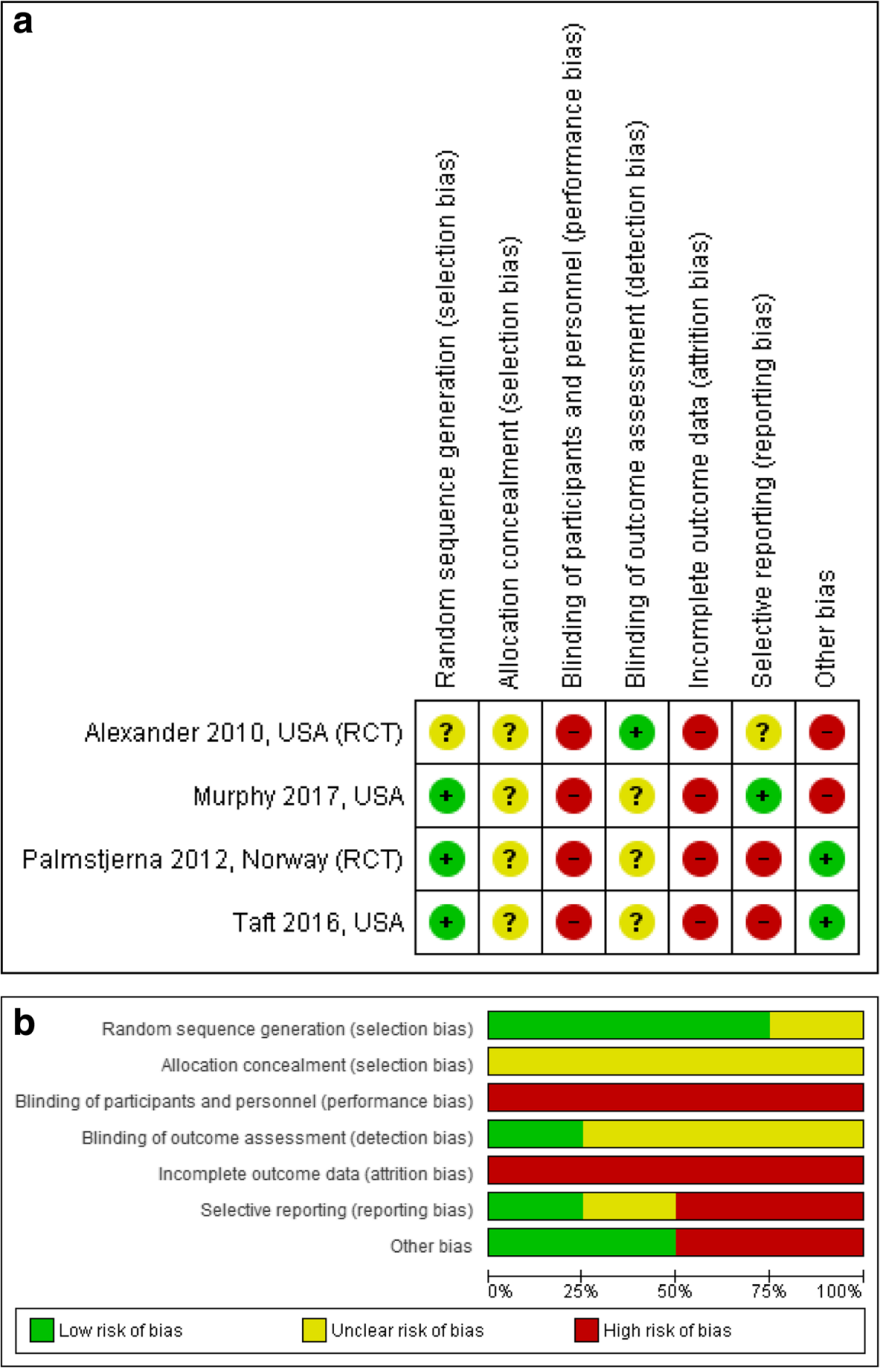
**a** Risk of bias summary: review authors’ judgements about each risk of bias item for each included study. **b** Risk of bias graph: review authors’ judgements about each risk of bias item for each included study

The risk-of-bias ratings for the randomized controlled trials are displayed in Fig. 2 a and b, depicted from RevMan [29]. All the included randomized trials in this review are judged as having poor quality. Additional file 2 shows more detailed information about the risk of bias ratings of each study.

Alexander et al. [37], Murphy et al. [38], Palmstierna et al. [39] and Taft et al. [40], score a high overall risk of bias in reporting according to the recommendations in the Cochrane Collaboration’s tool for assessing risk of bias in randomised trials [27]. Alexander et al. [37] provide unclear information about the random sequence generation process, while the other three randomized controlled trials score a low risk of bias due to a detailed description of the random sequence generation. In all the four studies, the allocation concealment scores indicate that the risk is unclear due to inadequate description. Neither the participants nor the personnel were blinded to the treatment conditions in the four studies and therefore scored as high risk, although the research assistants making follow-up phone calls to the partners were blinded to the condition in the study by Alexander et al. [37].

With regard to incomplete data, two of the studies present no intention-to-treat analyses [37, 39]. While Murphy et al. [38], Palmstierna et al. [39] and Taft et al. [40] describe the distribution of attrition across groups, Alexander et al. [37] do not (they refer to another publication based on the same study). All four studies score as having high risk of bias for this item.

The risk of bias due to selective reporting is mixed across the studies. We found no protocol information on ClinicalTrials.gov for either Palmstierna et al. [39] or Alexander et al. [37]. Alexander et al. [37] only report on subjects completing the intervention and score at unclear risk of bias. Regarding other bias in the study of Alexander et al. [37], there is no power calculation or description of the how data were analysed and we suspect low statistical power. Palmstierna et al. [39] presents the results from self-reports of outcomes using the CTS and the associated *p*-values. However, the study only reports per-protocol results and gives no estimates of differences in reduced violence between the groups, hence this study is at high risk of bias on this domain. Taft et al. [40] did not report on the pre-defined secondary outcomes as stated in the Clinical Trials register, and score at high risk of bias on this domain. Murphy et al. [38] report all expected primary outcomes in the pre-specified way stated in the Clinical Trials register, and hence score at low risk of bias. With regard to other bias in the study of Murphy et al. [38], the imbalanced lack of compliance with allocated interventions between groups could cause bias and low statistical power. Hence, the study is at high risk of bias on this domain.

With respect to other sources of bias, Palmstierna et al. [39] were funded through the authors’ employment at St. Olav’s University Hospital and the Norwegian University of Science and Technology. Alexander et al. [37] were supported by the National Institute of Justice Grant. Murphy et al. [38] was funded by a grant from the National Institutes of Health, and Taft et al. [40] was supported by grants from the Department of Veterans Affairs and Department of Defence and through the use of the facilities and resources of the Providence Veterans Affairs Medical Center.

One of the non-randomized studies [41] is judged as having an overall moderate risk of bias, while the second study as having an overall serious risk of bias [42] (Table 3) according to ROBINS-I [28]. With regard to bias due to confounding, the study by Haggård et al. [41] statistically controlled for baseline recidivism risk that might have confounded the association between treatment status and recidivism in violent behaviour. The study by Boira et al. [42] scores as having low risk of bias due to confounding since the participants were selected from the target population and the study controlled for possible baseline confounding. Furthermore, the four groups had comparable sociodemographic characteristics.

**Table 3:** Risk of Bias summary according to ROBINS-I in the non-randomized studies

1^st^author, year, study design	Bias due to confounding	Bias in selection of participants into the study	Bias in classification of interventions	Bias due to deviations from intended interventions	Bias due to missing data	Bias in measurement of outcomes	Bias in selection of the reported results	Overall bias
Haggård, 2015, Sweden, Controlled cohort retrospective study [31]	Low risk Controlled statistically for baseline recidivism risk that might confound the association between treatment status and recidivism	Low risk All participants who would have been eligible for the target trial were included in the study	Low risk The intervention group (IG) and control group (CG) are clearly defined. Information about intervention status was obtained retrospectively	Low risk Followed an Intention-To-Treat (ITT)-approach. Other co-interventions that might have affected the outcome were balanced across IG and CG. The assessor extracting data was blinded to recidivism data	Low risk Attrition from treatment was described (IG 27%). The study had complete outcome measurement based on registry information	Low risk Retrospective study with already reported outcomes	Moderate Performed the study after the intervention was finished. There is no published protocol, making it difficult to know if the outcomes were pre-defined	Moderate
Boira,2013, Spain, Quasi-experimental study [2]	Low risk The participants were selected from the target population. The study had controlled for possible baseline confounding, and the three groups are comparable for sociodemographic characteristics	Low risk All participants who would have been eligible for the target trial were included in the study	Low risk The four groups are clearly defined	No information Insufficient information with respect to ITT-analysis and adherence to interventions	Moderate There is unclear information about recidivism data on the intervention groups at 18-months follow up (the outcome is presented as total participants, *N* = 44, making it impossible to separate the effects between the four groups status). Low attrition. It is unclear how missing data was analysed	Moderate Lack of blind outcome assessments. The methods of outcome assessment were comparable across three of the four groups for pre- and post-assessments. Errors of measurement occurs at 18-months follow-up (non-differential measurements are presented with respect to conviction)	Serious There is no published protocol, making it difficult to know if the outcomes were pre-defined The lack of differentiation between treatment modalities in presenting the results at 18-month follow-up makes it difficult to judge whether the observed effect is associated to group treatment	Serious

Both studies are judged as having low risk of bias in the selection of participants for the study since both include all participants eligible for the target trial. Both studies clearly define the intervention and control groups and score as low risk on bias in the classification of interventions. Haggård et al. [41] followed an intention-to-treat approach and are therefore judged as having low risk of bias due to deviations from intended interventions. Boira et al. [42] provided insufficient information about intention-to-treat analysis, adherence to the interventions and about the control group outcomes at post-test, hence there is no information to judge this item.

Risk of bias due to missing data is judged as low for Haggård et al. [41] since the study is retrospective and based on register data. Hence, no attrition from the study would affect the outcome. Furthermore, the study provides complete outcome measurements based on registry information. In the study of Boira et al. [42] there is insufficient information to judge this item. They report low attrition from the study but provide unclear information on missing data besides that.

The retrospective study by Haggård et al. [41] is judged as having low risk of bias in the measurement of outcomes since the results were already reported and the methods of outcome assessment were comparable across the intervention and control groups. Moreover, one assessor was blinded to recidivism data on any crime conviction in the past 5 years, any previous conviction of IPV, any previous conviction of a sexual offense, young age (below 21) at first known crime, any previous conviction of violation of a restraining order, current abuse or dependence on alcohol or drugs. The study by Boira et al. [42] is judged as having moderate risk of bias on this item due to a lack of blind outcome assessments and unclear information on the outcomes and intervention status for 18 months of follow up (the outcome is presented as the total participants (*N* = 44), making it impossible to separate the effects between the status of the four groups).

No published protocol was found for either of the studies [41, 42], making it difficult to determine whether the outcomes were predefined. Also, in the Boira [42] study a lack of differentiation between treatment modalities in presenting the results at 18-month follow-up makes it difficult to judge whether the observed effect is associated with group treatment. The treatment programs used by Boira et al. [42] (personal communication) were not compared, and they instead measured the effect of each program separately on new reports of intimate partner violence.

With respect to other sources of bias, Haggård et al. [41] reported indirect funding from the Swedish Prison and Probation Service through the authors’ employment there. Boira et al. [42] did not report funding but had a collaboration agreement between the General Secretariat of Penitentiary Institutions and the College of Psychologists of Aragon.

### Primary outcome: Effect on violent behaviour

The reported primary and secondary outcomes are summarized narratively given the considerable diversity of how they were assessed and the report of data in the studies included. Tables 1 and 2 display the primary outcome measures. Four randomized controlled trials [37–40] including 731 clients and 202 partners, and two non-randomized studies [41, 42] including 854 clients report outcomes on violent behaviour. Four studies [37–40] assessed violent behaviour using the Conflict Tactics Scale (CTS/CTS2) [43]. One study obtained register data from the Swedish Prison and Probation Service and court records on reconviction for violent crime against an intimate partner [41], while another study used register data on intimate partner violence reported to the police [42]. Only one study addressed sexual violence [41].

The small study by Palmstierna et al. [39] (*N* = 26) indicates a protective effect of CBGT on self-reported violence related to intimate partners as compared to the waitlist control, immediately after the intervention. This study also finds a significant correlation between low age and continued physically violent behaviour. The substantially larger study by Alexander et al. [37] (*N* = 528) find no differences with respect to perpetrator self-reports of violence at the end of treatment between men assigned to a group treatment program based on the stages of change model and motivational interviewing (SOCMI) and those in a program based on the Duluth model-inspired CBT. Of the 43% of partners who responded, fewer partners in the SOCMI group reported having experienced physical aggression at follow-up. Murphy et al. [38] (*N* = 42) find that cognitive behaviour group therapy produces outcomes equal to or better than individual cognitive behaviour therapy. The difference between the two conditions are statistically significant for partner reports of psychological violence and exceed a medium effect size for physical assaults and emotional abuse. Taft et al. [40] (*N* = 135) report that the intervention was more effective than the control condition in reducing psychological and physical intimate partner violence, with a small-to-medium between-group effect size.

Haggård et al. [41] report that 19% i.e. 65 of the 340 participants in the treatment group and 19% i.e. 84 of the 452 controls recidivated in violence against a partner or former partner during follow-up. In the small study by Boira et al. [42] (*N* = 65, four different comparison groups), the authors conclude that they cannot obtain any conclusive evidence on any of the many outcome measures. As for their primary outcome (police reports after 18 months on new intimate partner violence), they do not compare the three programs. Furthermore, they do not report differences between the programs and the control group, and 94% (*N* = 44) of the program participants (regardless of study condition) did not have any new incidents of intimate partner violent reported to the police at 18-month follow-up. The primary outcome for the control group is not reported.

### Secondary outcomes

None of the studies included report treatment effects on physical health, quality of life, emotional regulation, and substance use after treatment. Only Boira et al. [42] report the effects of treatment on mental health as measured by the Symptom Checklist-90 (SCL-90). They found lower scores after structured group therapy in the SCL-90 depression dimension, Global Severity Index, and total symptom load.

### Other measurements

Two studies [37, 42] assess the participants’ readiness to change using The University of Rhode Island Change Assessment (URICA) [44]. However, the use of URICA by Boira et al. [42] is not satisfactorily explained. Alexander et al. [37] report a different outcome on physical violence: those with high initial readiness to change benefit more from group CBT than those with low initial readiness to change.

One study [42] assess empathy using the Spanish version of the Interpersonal Reactivity Index [45, 46], as well as hostility measured by the Spanish version of the Buss-Durkee Hostility Inventory [47, 48]. One study [37] assess risk factors for repeated violence using 12 items of the Danger Assessment Scale (DAS) [49]. Two studies [38, 40] use The Multidimensional Measure of Emotional Abuse (MMEA) [50] as an additional measure to assess psychological intimate partner violence. One study [38] assess relationship adjustment using the Dyadic Adjustment Scale (DAS) [51], the participants’ communication difficulties by partner reports on the Spouse Verbal Problem Checklist [52], and the participants’ responses to challenging relationship scenarios using the Articulated Thoughts in Simulated Situations [53] paradigm.

## Discussion

This systematic review evaluates and updates the evidence published on the effectiveness of cognitive behavioural group therapy for male perpetrators of intimate partner violence since the Cochrane review on this topic published in 2007 and replicated in 2011 [16, 23]. Only six studies met our inclusion criteria. Our main finding supports the results of the last updated review by Smedslund et al. [23] in 2011, that the evidence for this therapy is still inconclusive.

Three of the included studies found a reduction in physical violence among participants in the group-based interventions [38–40]. However, these studies were small and most of the findings relied solely on self-report from the perpetrators. The larger study by Alexander et al. [37] included 528 male participants and found only marginal differences in self-reported violence with respect to the type of treatment. Boira et al. [42] relied on police reports, which are known to capture only a small part of the actual incidents of intimate partner violence. Furthermore, the participants were not randomly selected for the different treatment modalities. Moreover, Boira et al. [42] had a wide range of outcomes without differentiation between primary and secondary outcomes. They reported only pre-post evaluations without comparing the group change differences. The study by Haggård et al. [41] reported the recurrence of intimate partner violence based on new convictions, which is also subject to the same limitation as the method by Boira et al. [42]: it does not necessarily show the true picture with regard to the violence that is actually occurring.

This review clearly confirms that self-reported outcomes like physical health, mental health, quality of life, emotional regulation and substance use are scarcely addressed when investigating the effectiveness of cognitive behavioural therapy for anger and aggressive behaviour. Future randomized controlled trials should therefore also address these outcomes.

A randomized controlled trial design is preferred when evaluating treatment effects due to confounding by indication. Nevertheless, only four of the six studies reviewed are randomized controlled trials. Furthermore, one of the studies has a limited sample size of only 26 participants [39].

When evaluating treatment effects, it is necessary to consider the treatment context [54, 55]. Delivering treatment within the prison service is different from an outpatient setting. The therapy is given in different settings across all the included studies, and most of the participants are involuntarily referred except for those examined in the study by Palmstierna et al. [39]. It is important to separate participants who are involuntarily assigned to treatment from those seeking treatment on their own initiative since they probably represent different subtypes of perpetrators with different associated risks of recurrent violence and treatment compliance [56, 57]. Earlier systematic reviews of cognitive therapy for perpetrators of intimate partner violence have not distinguished sufficiently between the different subtypes of perpetrators and the type of contexts where the treatment is delivered.

### Limitations and implications for future research

Since only six studies met the inclusion criteria, the conclusions drawn from this review should be interpreted with caution. We found reasons to suspect that there is a high risk of bias across the included studies, mainly due to lack of blinding and incomplete outcome data reporting. Also, allocation concealment and other important domains were poorly reported and represents a threat to the certainty of the overall evidence.

Two of the studies used register data on convictions and police reports, and four studies used self- and/or partner reports. The lack of standardisation of the study design, follow-up time, and outcome measurement found in the included studies prevents us from performing a meaningful meta-analysis. Not reporting the outcomes according to the original random assignment violates the intention behind random assignment and makes the experiment less likely to take into account possible confounding by indication.

The scarce evidence on the effect of group-based CBT calls for well-conducted randomised controlled trials in different settings, as well as different and defined selections of perpetrators. The findings of this review underscore these important areas for future research, which is in line with earlier evidence on different treatment modalities for perpetrators of intimate partner violence [23, 58–60]. Our review and previous research on intimate partner violence programmes reveal that a combination of multiple theoretical models and treatment modalities are common in clinical practice, which makes outcome evaluations challenging [55, 61]. In future research, the elements of the treatment should at least be described clearly to make it possible to evaluate and compare treatment effects [54]. It is also important to ascertain the therapeutic adherence to the protocol, which will increase the attribution of effects or lack of effects to the intervention. Furthermore, non-randomized studies should publish protocols including a pre-analysis plan. It is also recommended that randomized controlled trials use [26] and follow the CONSORT guidelines [62, 63].

## Conclusion

The evidence is still inconclusive with regard to the effectiveness of group-based CBT in reducing violence from men towards their female partners – a situation that is due to a lack of high-quality randomized controlled trials on the subject. An important implication for future research in this area is to put an emphasis on describing the interventions in detail and reporting the study design and finally, how the study was carried out.

Our review also reveals that, so far, few studies have investigated how group-based CBT affects self-reported outcomes on physical health, mental health, quality of life, emotional regulation, substance use, and socioeconomic outcome among perpetrators. Based on our findings, future studies should adopt a randomized controlled study design with clear criteria for randomization, blinding and allocation concealment. Reduction of violent behaviour should be the primary outcome, as measured by both self-reports and partner-reports. A clear description of the investigated perpetrator population is warranted since studies of convicted perpetrators should be separated from studies of non-convicted.

## Additional files

Additional file 1: Search strategies. A detailed description of the search strategies and the used search terms. (DOCX 19 kb)

Additional file 2: Risk of Bias Ratings. A detailed description of the risk of bias ratings of the included randomized controlled trials. (DOCX 20 kb)

## Abbreviations

CAPS: Clinician-Administered PTSD Scale
CBGT: Cognitive Behavioural Group Therapy
CBT: Cognitive Behavioural Therapy
CG: Control Group
CTS: Conflict Tactics Scale extended version
CTS2: Conflict Tactics Scales–Revised
DAS: Danger Assessment Scale
ICBT: Individual Cognitive Behavioural Therapy
IDAP: Integrated Domestic Abuse Program
IG: Intervention Group
IPV: Intimate Partner Violence
ITT: Intention-To-Treat
MINI: Mini-International Neuropsychiatric Interview
MMEA: Multidimensional Measure of Emotional Abuse
PRISMA: Preferred Reporting Items for Systematic Reviews and Meta-Analyses
PROSPERO: Prospective Register of Systematic Reviews
RCT: Randomized Controlled Trial
RevMan: Review Manager
RoB: Risk of Bias
ROBINS-I: Risk of Bias In Non-Randomized Studies of Interventions tool
SCL-90: Symptom Checklist-90
SOCMI: Stages Of Change model and Motivational Interviewing
TIDIeR: Template for Intervention Description and Replication
URICA: University of Rhode Island Change Assessment
