# Supplementary material for: Cognitive behavioural group therapy for male perpetrators of intimate partner violence: a systematic review
Source: BMC Psychiatry. 2019 Jan 8;19:11. doi: 10.1186/s12888-019-2010-1 (PMC6325780; doi:10.1186/s12888-019-2010-1)
Supplement: Supplementary file 1 — Search strategies. A detailed description of the search strategies and the used search terms. (DOCX 19 kb) [file 12888_2019_2010_MOESM1_ESM.docx]

**Additional file 1:** Search strategies

Bibliography of Nordic Criminology

Bibliography of Nordic criminology (<http://www.nsfk.org/>) was searched using the terms; ("domestic violence" OR "battered women" OR "family violence" OR "partner violence") AND therap*

CINAHL

S29 S8 AND S28 Limiters - Published Date: 20100101-20180212

S28 S8 AND S28

S27 S8 OR S9 OR S10 OR S11 OR S12 OR S13 OR S14 OR S15 OR S16 OR S17 OR S18 OR S19 OR S20 OR S21 OR S22 OR S23 OR S24 OR S25 OR S26

S26 TX ((key or core) N2 belief*)

S25 TX reattribution*

S24 TX (nat or nats)

S23 TX (automatic N2 (thought* or process*))

S22 TX (dysfunctional N2 (thought* or assumption* or rule* or appraisal* or belief* or attitude* or scheme*))

S21 TX (socratic* N3 (question* or method* or dialogue* or strateg* or sequence*))

S20 TX ((cognitiv* or mental*) N3 (map* or model))

S19 (MH "Concept Mapping")

S18 TX imager*

S17 (MH "Guided Imagery")

S16 (MH "Imagination+")

S15 TX (schema* or schemata*)

S14 TX cbt

S13 TX (rational* N3 emotive*)

S12 TX (behavio?r* N3 (therap* or train* or modif* or experiment*))

S11 TX (cognitiv* N3 (therap* or train* or techni* or question* or approach* or assessment*))

S10 (MH "Behavior Modification+")

S9 (MH "Cognitive Therapy+")

S8 (MH "Behavior Therapy+")

S7 S1 OR S2 OR S3 OR S4 OR S5 OR S6 OR S7

S6 TX ((male* or men or man or partner* or spouse* or husband or fiance or cohabitant* or live?in) N3 (batter* or perpetrator* or abus* or violen* or beat* or assault))

S5 TX ((abus* or batter* or beat* or assault*) N3 (wom?n or partner* or spouse* or female* or wife or wives or domestic* or fiance or cohabitant* or live?in))

S4 TX ((familiy or domestic or conjugal or partner) N3 violence).tw.

S3 (MH "Intimate Partner Violence")

S2 (MH "Domestic Violence+")

S1 (MH "Battered Women")

Embase via Ovid Embase <1974 to 2018 February 12>

1 exp domestic violence/ or battered woman/ or family violence/ or partner violence/

2 ((familiy or domestic or conjugal or partner) adj3 violence).tw.

3 ((abus$ or batter$ or beat$ or assault$) adj3 (wom?n or partner$ or spouse$ or female$ or wife or wives or domestic$ or fiance or cohabitant$ or live?in)).tw.

4 ((male$ or men or man or partner$ or spouse$ or husband or fiance or cohabitant$ or live?in) adj3 (batter$ or perpetrator$ or abus$ or violen$ or beat$ or assault)).tw.

5 or/1-4

6 behavior therapy/ or cognitive therapy/

7 behavior modification/

8 (cognitiv$ adj3 (therap$ or train$ or techni$ or question$ or approach$ or assessment$)).tw.

9 (behavio?r$ adj3 (therap$ or train$ or modif$ or experiment$)).tw.

10 (rational$ adj3 emotive$).tw.

11 cbt.tw.

12 (schema$ or schemata$).tw.

13 imagery/

14 imager$.tw.

15 ((cognitiv$ or mental$) adj3 (map$ or model)).tw.

16 (socratic$ adj3 (question$ or method$ or dialogue$ or strateg$ or sequence$)).tw.

17 (dysfunctional adj2 (thought$ or assumption$ or rule$ or appraisal$ or belief$ or attitude$ or scheme$)).tw.

18 (automatic adj2 (thought$ or process$)).tw.

19 (nat or nats).tw.

20 reattribution$.tw.

21 ((key or core) adj2 belief$).tw.

22 or/6-21

23 5 and 22

24 limit 23 to yr="2010 -Current"

ERIC (Educational Resources Information Center) via ProQuest

(SU.EXACT(family violence) OR SU.EXACT(battered women) OR ti,ab(abuse* NEAR/3 (wom?n OR partner* OR spouse* OR female* OR wife OR wives or domestic*)) OR ti,ab(batter* NEAR/3 (wom?n OR partner* OR spouse* OR female* OR wife OR wives)) OR ti,ab(violen* NEAR/3 (partner* OR spous* OR family OR families OR domestic* OR conjugal*))) AND (SU.EXACT(cognitive restructuring) SU.EXACT(behavior modification) OR ti,ab(cognitive* NEAR/3 (therap* or train*)) OR ti,ab(behavio?r* NEAR/3 (therap* OR train*)) OR ti,ab(behavio?r* NEAR/3 modif*)) AND pd(>20091231)

Grey literature report

The Grey literature report (http://greylit.org) was searched using the terms; domestic violence AND therapy.

MEDLINE via Ovid (Ovid MEDLINE(R) ALL <1946 to February 12, 2018>)

1 Battered Women/

2 domestic violence/ or spouse abuse/

3 ((familiy or domestic or conjugal or partner) adj3 violence).tw.

4 ((abus$ or batter$ or beat$ or assault$) adj3 (wom?n or partner$ or spouse$ or female$ or wife or wives or domestic$ or fiance or cohabitant$ or live?in)).tw.

5 ((male$ or men or man or partner$ or spouse$ or husband or fiance or cohabitant$ or live?in) adj3 (batter$ or perpetrator$ or abus$ or violen$ or beat$ or assault)).tw.

6 or/1-5

7 behavior therapy/ or cognitive therapy/

8 psychotherapy, rational-emotive/

9 (cognitiv$ adj3 (therap$ or train$ or techni$ or question$ or approach$ or assessment$)).tw.

10 (behavio?r$ adj3 (therap$ or train$ or modif$ or experiment$)).tw.

11 (rational$ adj3 emotive$).tw.

12 cbt.tw.

13 (schemas or schematas).tw.

14 "Imagery (Psychotherapy)"/

15 imager$.tw.

16 ((cognitive$ or mental$) adj3 (map$ or model$)).tw.

17 (socratic$ adj3 (question$ or method$ or dialogue$ or strateg$ or sequence$)).tw.

18 (dysfunctional adj2 (thought$ or assumption$ or rule$ or appraisal$ or belief$ or attitude$ or scheme$)).tw.

19 (automatic adj3 (thought$ or process$)).tw.

20 (nat or nats).tw.

21 reattribution$.tw.

22 ((key or core) adj2 belief$).tw.

23 or/7-22

24 6 and 23

25 limit 24 to yr="2010 -Current"

Open Grey search strategy

Open Grey (http://www.opengrey.eu/search/). All indexed fields or all non-indexed fields were searched for the terms; ("domestic violence" OR "battered women" OR "family violence" OR "partner violence" OR "spouse abuse" OR “spousal abuse”) AND therapy.

PsycINFO via Ovid (PsycINFO <1987 to February Week 1 2018>)

1 partner abuse/

2 family violence/

3 ((familiy or domestic or conjugal or partner) adj3 violence).tw.

4 battered females/

5 ((abus$ or batter$ or beat$ or assault$) adj3 (wom?n or partner$ or spouse$ or female$ or wife or wives or domestic$ or fiance or cohabitant$ or live?in)).tw.

6 ((male$ or men or man or partner$ or spouse$ or husband or fiance or cohabitant$ or live?in) adj3 (batter$ or perpetrator$ or abus$ or violen$ or beat$ or assault)).tw.

7 or/1-6

8 cognitive therapy/

9 cognitive behavior therapy/

10 rational emotive behavior therapy/

11 exp behavior therapy/

12 behavior modification/

13 cognitive assessment/

14 (cognitiv$ adj3 (therap$ or train$ or techni$ or question$ or approach$ or assessment$)).tw.

15 (behavio?r$ adj3 (therap$ or train$ or modif$ or experiment$)).tw.

16 (rational$ adj3 emotive$).tw.

17 cbt.tw.

18 schema/

19 (schema$ or schemata$).tw.

20 exp imagery/ or conceptual imagery/

21 imager$.tw.

22 cognitive maps/

23 mental models/

24 ((cognitiv$ or mental$) adj3 (map$ or model)).tw.

25 (socratic$ adj3 (question$ or method$ or dialogue$ or strateg$ or sequence$)).tw.

26 (dysfunctional adj2 (thought$ or assumption$ or rule$ or appraisal$ or belief$ or attitude$ or scheme$)).tw.

27 (automatic adj2 (thought$ or process$)).tw.

28 (nat or nats).tw.

29 reattribution$.tw.

30 ((key or core) adj2 belief).tw.

31 or/8-29

32 7 and 31

33 limit 32 to yr="2010 -Current"

The Cochrane Library (CENTRAL, Cochrane Reviews, DARE)

#1 MeSH descriptor: [Battered Women] explode all trees

#2 MeSH descriptor: [Domestic Violence] explode all trees

#3 MeSH descriptor: [Spouse Abuse] explode all trees

#4 ((familiy or domestic or conjugal or partner*) near/3 violence):ti,ab,kw

#5 ((abus* or batter* or beat* or assault*) near/3 (wom*n or partner* or spouse* or female* or wife or wives or domestic* or fiance or cohabitant* or live-in)):ti,ab,kw

#6 ((male* or men or man or partner* or spouse* or husband or fiance or cohabitant* or live-in) near/3 (batter* or perpetrator* or abus* or violen* or beat* or assault)):ti,ab,kw

#7 #1 or #2 or #3 or #4 or #5 or #6

#8 MeSH descriptor: [Behavior Therapy] explode all trees

#9 MeSH descriptor: [Cognitive Therapy] explode all trees

#10 MeSH descriptor: [Psychotherapy, Rational-Emotive] explode all trees

#11 (cognitive* near/3 (therap* or train* or techni* or question* or approach* or assessment*)):ti,ab,kw

#12 ((behavior* or behaviour*) near/3 (therap* or train* or modif* or experiment*)):ti,ab,kw

#13 (rational* near/3 emotive*):ti,ab,kw

#14 (cbt):ti,ab,kw

#15 (schemas or schematas):ti,ab,kw

#16 MeSH descriptor: [Imagery (Psychotherapy)] explode all trees

#17 (imager*):ti,ab,kw

#18 ((cognitive* or mental*) near/3 (map* or model*)):ti,ab,kw

#19 (Socratic* near/3 (question* or method* or dialogue* or strateg* or sequence*)):ti,ab,kw

#20 (dysfunctional near/2 (thought* or assumption* or rule* or appraisal* or belief* or attitude* or scheme*)):ti,ab,kw

#21 (automatic near/3 (thought* or process*)):ti,ab,kw

#22 (nat or nats):ti,ab,kw

#23 (reattribution*):ti,ab,kw

#24 ((key or core) near/2 belief*):ti,ab,kw

#25 #8 or #9 or #10 or #11 or #12 or #13 or #14 or #15 or #16 or #17 or #18 or #19 or #20 or #21 or #22 or #23 or #24

#26 #7 and #25 Publication Year from 2010 to 2018

SCOPUS

TITLE-ABS-KEY ( ( ( familiy OR domestic OR conjugal OR partner ) W/3 violence ) OR ( ( abus* OR batter* OR beat* OR assault* ) W/3 ( wom?n OR partner* OR spouse* OR female* OR wife OR wives OR domestic* OR fiance OR cohabitant* OR live?in ) ) OR ( ( male* OR men OR man OR partner* OR spouse* OR husband OR fiance OR cohabitant* OR live?in ) W/3 ( batter* OR perpetrator* OR abus* OR violen* OR beat* OR assault ) ) ) AND ( ( cognitiv* W/3 ( therap* OR train* OR techni* OR question* OR approach* OR assessment* ) ) OR ( behavio?r* W/3 ( therap* OR train* OR modif* OR experiment* ) ) OR ( rational* W/3 emotive* ) OR cbt OR schemas OR schematas OR imager* OR ( ( cognitive* OR mental* ) W/3 ( map* OR model* ) ) OR ( socratic* W/3 ( question* OR method* OR dialogue* OR strateg* OR sequence* ) ) OR ( dysfunctional W/2 ( thought* OR assumption* OR rule* OR appraisal* OR belief* OR attitude* OR scheme* ) ) OR ( automatic W/3 ( thought* OR process* ) ) OR nat OR nats OR reattribution* OR ( ( KEY OR core ) W/2 belief* ) ) AND ( INDEXTERMS ( "clinical trials" OR "clinical trials as a topic" OR "randomized controlled trial" OR "Randomized Controlled Trials as Topic" OR "controlled clinical trial" OR "Controlled Clinical Trials" OR "random allocation" OR "Double-Blind Method" OR "Single-Blind Method" OR "Cross-Over Studies" OR "Placebos" OR "multicenter study" OR "double blind procedure" OR "single blind procedure" OR "crossover procedure" OR "clinical trial" OR "controlled study" OR "randomization" OR "placebo" ) OR TITLE-ABS-KEY ( "clinical trials" OR "clinical trials as a topic" OR "randomized controlled trial" OR "Randomized Controlled Trials as Topic" OR "controlled clinical trial" OR "Controlled Clinical Trials as Topic" OR "random allocation" OR "randomly allocated" OR "allocated randomly" OR "Double-Blind Method" OR "Single-Blind Method" OR "Cross-Over Studies" OR "Placebos" OR "cross-over trial" OR "single blind" OR "double blind" OR "factorial design" OR "factorial trial" ) OR TITLE-ABS ( trial* OR rct* OR random* OR blind* OR control* OR sham ) ) AND PUBYEAR > 2009

Social Care Online

Social Care Online (<http://www.scie-socialcareonline.org.uk/>) was searched using the terms; “domestic violence” AND therapy.

Sociological Abstracts via ProQuest

S27 S7 and S26 and pd(>20091231)

S26 S8 or S9 or S10 or S11 or S12 or S13 or S14 or S15 or S16 or S17 or S18 or S19 or S20 or S21 or S22 or S23 or S24 or S25

S25 ti,ab((key or core) NEAR/1 belief*)

S24 ti,ab(reattribution*)

S23 ti,ab(nat or nats)

S22 ti,ab(automatic NEAR/1 (thought* or process*))

S21 ti,ab(dysfunctional NEAR/1 (thought* or assumption* or rule* or appraisal* or belief* or attitude* or scheme*))

S20 ti,ab(socratic* NEAR/3 (question* or method* or dialogue* or strateg* or sequence*))

S19 ti,ab((cognitiv* or mental*) NEAR/3 (map* or model))

S18 SU.EXACT("Cognitive Mapping")

S17 ti,ab(imager*)

S16 SU.EXACT("Images" or "Mass Media Images")

S15 ti,ab(schema* or schemata*)

S14 ti,ab(cbt)

S13 ti,ab(rational* NEAR/3 emotive*)

S12 ti,ab(behavio?r* NEAR/3 (therap* or train* or modif* or experiment*))

S11 ti,ab(cognitiv* NEAR/3 (therap* or train* or techni* or question* or approach* or assessment*))

S10 SU.EXACT("Treatment Methods")

S9 SU.EXACT("Treatment Programs")

S8 SU.EXACT("Behavior Modification")

S7 S1 or S2 or S3 or S4 or S5 or S6

S6 ti,ab((male* or men or man or partner* or spouse* or husband or fiance or cohabitant* or live?in) NEAR/3 (batter* or perpetrator* or abus* or violen* or beat* or assault))

S5 ti,ab((abus* or batter* or beat* or assault*) NEAR/3 (wom?n or partner* or spouse* or female* or wife or wives or domestic* or fiance or cohabitant* or live?in))

S4 ti,ab((familiy or domestic or conjugal or partner) NEAR/3 violence)

S3 SU.EXACT("Family Violence")

S2 SU.EXACT("Spouse Abuse")

S1 SU.EXACT("Spouse Abuse")
