# Supplementary material for: Cognitive behavioural group therapy for male perpetrators of intimate partner violence: a systematic review
Source: BMC Psychiatry. 2019 Jan 8;19:11. doi: 10.1186/s12888-019-2010-1 (PMC6325780; doi:10.1186/s12888-019-2010-1)
Supplement: Supplementary file 2 — Risk of Bias Ratings. A detailed description of the risk of bias ratings of the included randomized controlled trials. (DOCX 20 kb) [file 12888_2019_2010_MOESM2_ESM.docx]

**Additional file 2:** Risk of Bias Ratings (RoB)

| **1^st^author,**  **year,**  **country** | **Random sequence generation** | **Allocation**  **concealment** | **Blinding**  **Participants** | **Blinding outcomes** | **Lost at follow-up**  **Incomplete data** | **Selective**  **Reporting** | **Other bias** | **Quality score** |
| --- | --- | --- | --- | --- | --- | --- | --- | --- |
| **Alexander, 2010, USA** | **Unclear**  The sequence generation process is not described | **Unclear**  No information about concealment | **High**  Participants and therapists were not blinded to treatment. The outcome is likely to be influenced by lack of blinding | **Unclear**  The study did not address whether the outcome assessors had knowledge of the allocated intervention  **Low**  Research assistants making partner follow-up phone calls were blinded | **High**  No intention-to-treat-analyses.  Attrition probability not described, but the authors referred to another paper from the same study. Confidence intervals not reported | **Unclear**  Not registered in the ClinicalTrials.gov.  Insufficient information to permit judgement of “low risk” or “high risk” | **High**  No power calculation and we suspect low statistical power. Logistic regression controls for (1) Language, Spanish or English; (2) Time between onset of treatment and victims report | **High RoB** |
| **Murphy, 2017, USA** | **Low**  Urn randomization | **Unclear**  No information about concealment | **High**  No indication of attempted blinding for participants or personnel. The outcome is likely to be influenced by lack of blinding | **Unclear**  No information on whether those performing the analyses of the data were blinded  For criminal justice data there is low risk of bias | **High**  1-3 cases excluded due to missing data in ICBT versus 6-9 cases in the CBGT | **Low**  Registered in the ClinicalTrials.gov (NCT00070863).  No indication of selective reporting of any of the outcomes. All expected outcomes were accounted for. The study protocol, as stated in the Clinical Trials register, contained the pre-defined primary outcomes. The outcomes were reported in the pre-specified way | **High**  15 of 21 received allocated intervention in CBGT versus all 21 received ICBT and we believe lack of compliance could cause bias.  Low statistical power | **High RoB** |
| **Palmstierna, 2012, Norway** | **Low**  Information about stratified randomisation by using block randomisation, but not how it was carried out | **Unclear**  No information about concealment | **High**  No blinding of the participants. The outcome is likely to be influenced by lack of blinding | **Unclear**  No information on whether those performing the analyses of the data were blinded | **High**  No intention-to-treat-analyses.  The study described the attrition probability: 5 in the intervention group and 4 in the waiting list group | **High**  Not registered in the ClinicalTrials.gov.  Only per-protocol results were presented.  Not estimated the differences in reduced violence between the groups, but *p*-value were reported | **Low**  No indication of other biases | **High RoB** |
| **Taft, 2016, USA** | **Low**  Information about randomisation by using block randomisation, using a random number generator | **Unclear**  No information about concealment | **High**  No blinding of the participants. The outcome is likely to be influenced by lack of blinding | **Unclear**  No information on whether those performing the analyses of the data were blinded | **High**  Intention-to-treat-analyses were performed.  The proportion of loss to follow-up is unevenly distributed between the intervention- and control group.  They have used a multilevel analysis, which under the assumption of missing at random are less susceptible for bias | **High**  Registered in the ClinicalTrials.gov (NCT01435512).  Did not report on all the pre-defined secondary outcomes as stated in the ClinicalTrials register.  Self-reports of pre-defined outcomes using CTS2 are reported.  Self-reports of perpetrator and partner were combined using CTS2.  95% confidence interval was reported | **Low**  No indication of other biases | **High RoB** |

*CTS2* Conflict Tactics Scales–Revised, *CBGT* Cognitive Behaviour Group Therapy, *ICBT* Individual Cognitive Behaviour Therapy
